# Effectiveness of H5P in improving student learning outcomes in an online tertiary education setting

**DOI:** 10.1007/s12528-023-09361-6

**Published:** 2023-03-16

**Authors:** Tarosh Jacob, Stephanie Centofanti

**Affiliations:** University of South Australia Online, UniSA City West Campus, Adelaide, South Australia 5000 Australia

**Keywords:** Adult learning, Distance education and online learning, Human–computer interface, Media in education, Teaching, Learning strategies

## Abstract

Innovative, pedagogically informed instructional design is instrumental in increasing student engagement and improving learning outcomes in online learning environments. Interactive learning resources provide students with the opportunity to engage with content in a more personalised manner. H5P (HTML 5 Package) is a collaborative platform that allows developers to create interactive content and has been regularly used in education settings. Some evidence suggests using interactive H5P resources in online education courses could lead to greater student engagement. However, to date, there has been little investigation into whether H5P resources can improve student learning outcomes. The current study aimed to assess whether using interactive H5P resources improved assessed learning outcomes in an online undergraduate psychology course. A randomized cross-over design was utilized to test whether students exposed to H5P interactive videos had improved assessment results when compared to a control group. This study found no meaningful differences in assessment scores between students exposed to H5P versus those that were not. There was low overall engagement with the interactive content. However, students who did engage with the resources reported a positive experience and indicated a preference for more interactive elements in future courses. Future research should extend on the instructional design obstacles identified in this study, for example, by examining whether improved accessibility and education on the benefits of interactive resources would increase engagement and grades.

## Introduction

Higher education institutions are fast moving towards digitizing their curriculums and offering courses in 100% online environments (Singh & Thurman, 2019). This move has increased educational opportunities for students who were previously unable to access university-level tertiary qualifications (Goodman et al., 2019; Stone, 2017). The move to a digital curriculum has been accelerated due to the COVID-19 pandemic that caused University campuses to shut down face-to-face classes and move to online teaching environments (Mishra et al., 2020). However, a common complaint among online students is that the online learning experience is isolating, which negatively affects learning outcomes, and contributes to an increase in student attrition rates (Bawa, 2016; Stone & Springer, 2019). Thus, it is important for universities to ensure that there are adequate supports in place in online learning environments. This could be in the form of innovative instructional design that increases student engagement with learning materials, and providing opportunities for students to reflect on their learning and seek feedback from teaching staff. The cognitivist theoretical approach posits that for learning to be effective, learning environments should be adaptive, personalized and in students’ control (Chatti et al., 2010). Similarly, the constructivist approach, posited by Doherty and Blake (2010) and Ellis and Goodyear (2013), calls for learning that is student centric, personalized and encourages self-regulation through deeper engagement with the content. Whilst there is no universal definition of personalized learning (Holmes et al., 2018), this paper conceptualizes personalized learning as technology-supported learning resources that adjust the pace of learning according to the needs of each student (Major & Francis, 2020). For example, interactive videos may use embedded pop quizzes that test student understanding of the content, and thus support students in deciding whether to proceed with the video or review the previous content before proceeding.

Bates (2015) describes the need for learning environments to be rich in content, whilst allowing students to develop and practice relevant skills that prepare them for an uncertain world. As an extension, Williams (2006) found that student success rates were tied to providing a variety of formative assessments and quality teacher-student interactions. Research has also indicated that personalized learning that actively engages students with the content improves student satisfaction and success. Furthermore when student satisfaction regarding course content and design is high, and students’ expectations of the online learning environment are exceeded, they are in turn more likely to engage further within the online learning environment (Cheng, 2014, 2019, 2020; Larsen et al., 2009; Lee, 2010; Lin & Wang, 2012).

These findings suggests that high quality interactive learning content would lead to better engagement with the content compared to non-interactive learning content. In addition to interactivity, Blieck et al. (2019) identified that learning environments that are personalized provide a space where students are able challenge themselves and go beyond the minimum level of knowledge reproduction, thereby improving learning outcomes. Educational technologies provide an opportunity for institutions to create course content that is interactive, personalized, and leads to tangible improvements in learning outcomes; especially if tied to strong pedagogy, student-centred and constructivist approaches, and peer feedback Wekerle et al., 2020; Lai & Bower, 2020).

One tool that allows educators to attempt to increase student engagement and academic achievement is H5P (HTML 5 Package). H5P is a free and open-sourced content collaboration framework that allows educators to create interactive content that can be embedded onto a variety of platforms including Moodle (moodle.com). H5P includes 39 open sourced, editable content applications such as interactive presentations, quizzes, interactive timelines, audio recordings, and flashcards (H5P.org). Interactions can be embedded into videos in an adaptive manner. Interactive presentations, through prompts embedded by the educator, allow students to test their understanding of content throughout the video rather than passively listening to it. For example, if a student responds incorrectly to a question embedded partway through an online video, they would be prompted to return to the section of the video explaining the particular concept. The adaptability afforded by H5P also creates a more personalised learning experience for the student, which Ellis and Goodyear (2013) call for in their constructivist approach to learning. The variety of applications allows for educators to follow the recommendations of Gikandi et al. (2011) and Williams (2006) that recommend inclusion of a variety of formative assessments as part of the instructional design of courses. H5P makes designing interactive materials easier for teachers and instructional designers, while possibly also improving student outcomes. Ploetzner’s (2022) recent meta-analysis found that enhanced interactive videos, such as those including questions and tasks, are more effective for retention and comprehension than videos without interactions. The interactive nature of H5P activities aims to engage students in what Ellis and Goodyear (2013) describe as, “learning through engaging in the metacognitive skills of reflection and self-regulation” (pg. 25).

The Interactive-Constructive-Active–Passive (ICAP) framework of cognitive engagement was developed by Chi and Wylie (2014). They hypothesized that increasingly complex engagement with learning materials is associated with deeper levels of learning. The ICAP framework categorizes four modes of engagement from least to most complex as *Passive, Active, Constructive* and finally *Interactive*. *Passive* engagement is when learners store new information in an isolated manner, such as listening to a lecture. Under the *Active* mode of engagement, learners are able to manipulate new information, which enables them to emphasize certain parts of the learning experience and integrate this with the new material, thereby forming stronger memory connections and easier retrieval. This is followed by the *Constructive* mode whereby learners are presented with conceptual material and asked to create inferences (induct, deduct and conclude), or integrate various previously learned material. The most complex is the *Interactive* mode of engagement, whereby learners participate in dialogue with peers, in which co-inferences create new knowledge pathways and thus a deeper understanding of material (Chi & Wylie, 2014).

Framed within ICAP, H5P applications allows for learners to be in the *Active* and *Constructive* modes of engagement, as they enable students to manipulate and reproduce their understanding of the learning material. H5P encompasses these principles by allowing the student to make decisions about their learning of the content, and thereby actively engage in the content in a meaningful manner. For example, relating to the *Active* mode of engagement, an interactive content video can be paused to incorporate a “fill-in-the-blank” activity (Appendix), which allows students to apply their understanding of presented concepts. Relating to the *Constructive* mode, H5P allows students to create inferences from theoretical concepts, and apply them to case study scenarios that can be assessed through multiple choice questions (Appendix) embedded within the video. These types of interactions that are designed to actively engage students’ cognitive processes have been found to be more effective than passive navigational features that simply allow students to navigate through a video (Ploetzner, 2022). The ability to control and interact through pausing allows the student the time to comprehend old content before new information is added. Having pauses in the video creates segmentation, which allows students to comprehend information in a logical, meaningful manner (Spanjers et al., 2010).

Thus, from a theoretical perspective, H5P should enable increased engagement and improve student outcomes. The incorporation of H5P activities should support a constructivist approach, whereby the range of interactive activities could facilitate the alignment of learning objectives and tasks with summative assessments, that is, what Biggs (1996) refers to as ‘Constructive Alignment’. Although a number of studies have shown that interactive videos can improve student engagement, only a handful of published studies exist on the effectiveness of H5P. Sinnayah et al. (2021) investigated the use of H5P presentations in physiology education and found that students who attempted the interactive activities (fill-in-the-blank and multiple choice questions) were consistently engaged with them, and 90% of students who engaged in the activities indicated that their level of content knowledge was significantly improved. Zeller et al. (2021) found that H5P provided opportunities to create stimulating discussion through the use of interactive videos. However, student outcomes were not measured in this report. Preliminary findings from a conference proceeding showed that when compared to previous cohorts, replacing face-to-face lectures with H5P interactives led to improved student pass and retention rates (Wilkie & Zakaria, 2017). All of these studies, however, appear to be observational in design or rely on self-report outcomes, which makes it difficult to take cohort effects into account. Given the accessibility and growing popularity of H5P on online learning platforms, assessment of the efficacy of this tool is currently somewhat lacking.

This study aims to build on the existing literature using an empirical research design that evaluates the effectiveness of H5P for improving student outcomes. More specifically, the aim of this study is to investigate whether an intervention of H5P interactive video features improve student learning outcomes in a summative assessment quiz. The first hypothesis is that students who complete interactive elements of an H5P video resource will have improved grades on an assessed quiz, compared to students who viewed a non-interactive video resource on the same topic. A second hypothesis, in line with previous studies, is that students would report positive subjective experiences of H5P resources.

## Material and methods

### Participants

The present study was situated in two offerings of a 100% online first-year undergraduate psychology course delivered by the University of South Australia (UniSA) Online in 2020. The course ran over 10 weeks with a new topic covered each week. The course was delivered asynchronously, with strategic interactions from the teaching team (e.g., scheduled forum posts 2–3 times per week). Therefore, students were expected to be self-directed as they navigated through the course content. In total, 572 students (Males = 101, Females = 469, Unspecified = 2; Age range 18–78y) participated in the study from January-November 2020. Students were enrolled in the online ‘Psychology Concepts’ course as part of 27 different degrees.

### Ethics

This study received ethics approval (Application 202,259) from the UniSA Human Research Ethics Committee. At the commencement of the course, students were provided with a written overview of the research study. They were informed that they would have access to the H5P resources and undertake the quiz as part of the course regardless of their participation in the research project, and that they were free to opt out if they did not want their data (quiz results) to be included in analyses. They were also informed that opting out would not detrimentally impact them, their course results, or their relationship with teaching staff in any way.

### Research design

Student experiences and results were collected in the following ways to triangulate the data:

1. Content knowledge acquired through formative standard content videos vs H5P interactive content videos was measured by analysing final grades on a summative assessment quiz (see 2.3.1), and;
2. Student experiences of engagement with the H5P interactive resources were measured via a voluntary anonymous student feedback survey instrument (see 2.3.2).

#### Summative assessment quiz intervention

In Week 7 of the course, students were required to undertake a summative assessment quiz that tested their knowledge of the course content. As part of their weekly studies and in preparation for the summative quiz, students were encouraged to complete readings and watch a number of content videos. For the purposes of the current study, the ‘Interactive Video’ H5P feature was utilized to incorporate interactive elements into two existing content videos. The interactive elements for both videos included: Multiple Choice Questions; Fill in the Blanks; Drag and Drop; Crossroads and Text activities (see Appendix). One interactive video was embedded in Week 4 of the course (weekly topic: Learning), and its learning objective was to explain the processes behind Classical Conditioning. The second interactive video was situated in Week 5 of the course (weekly topic: Sleep and Shiftwork), and its learning objective was to explain the circadian and homeostatic systems involved in Sleep Regulation. The interactive elements were positioned at key points of each content video to test student understanding of the content being presented. For example, an interactive element such as a fill in-the-blanks activity was incorporated immediately following the explanation of a sub-topic within the video. This enabled a pause in the video where students could apply their learning before proceeding to the next sub-topic within the video (see Appendix). The interactive elements also provided an opportunity for students to reflect on their learning progress before attempting the summative assessment quiz in Week 7. The summative assessment quiz had 20 questions in total, which included 5 questions on Classical Conditioning and 5 questions on Sleep Regulation. The other 10 questions assessed content from the rest of the week and are not included in this study.

Participating students were randomly allocated into two groups: Group A (n = 277) and Group B (n = 295). Each group received access to one of the two interactive H5P videos prior to completing the summative quiz in Week 7:

Group A: Access to the H5P Interactive Video resource on Classical Conditioning in Week 4; access to the original non-interactive video on the Sleep Regulation in Week 5.

Group B: Access to the original non-interactive video on Classical Conditioning; access to the H5P Interactive Video on the Sleep Regulation.

This between-groups design was implemented to allow for comparisons between summative assessment quiz grades in students who engaged with the interactive version of the content video, compared to those who only had access to a non-interactive content video.

**Fig. 1:**
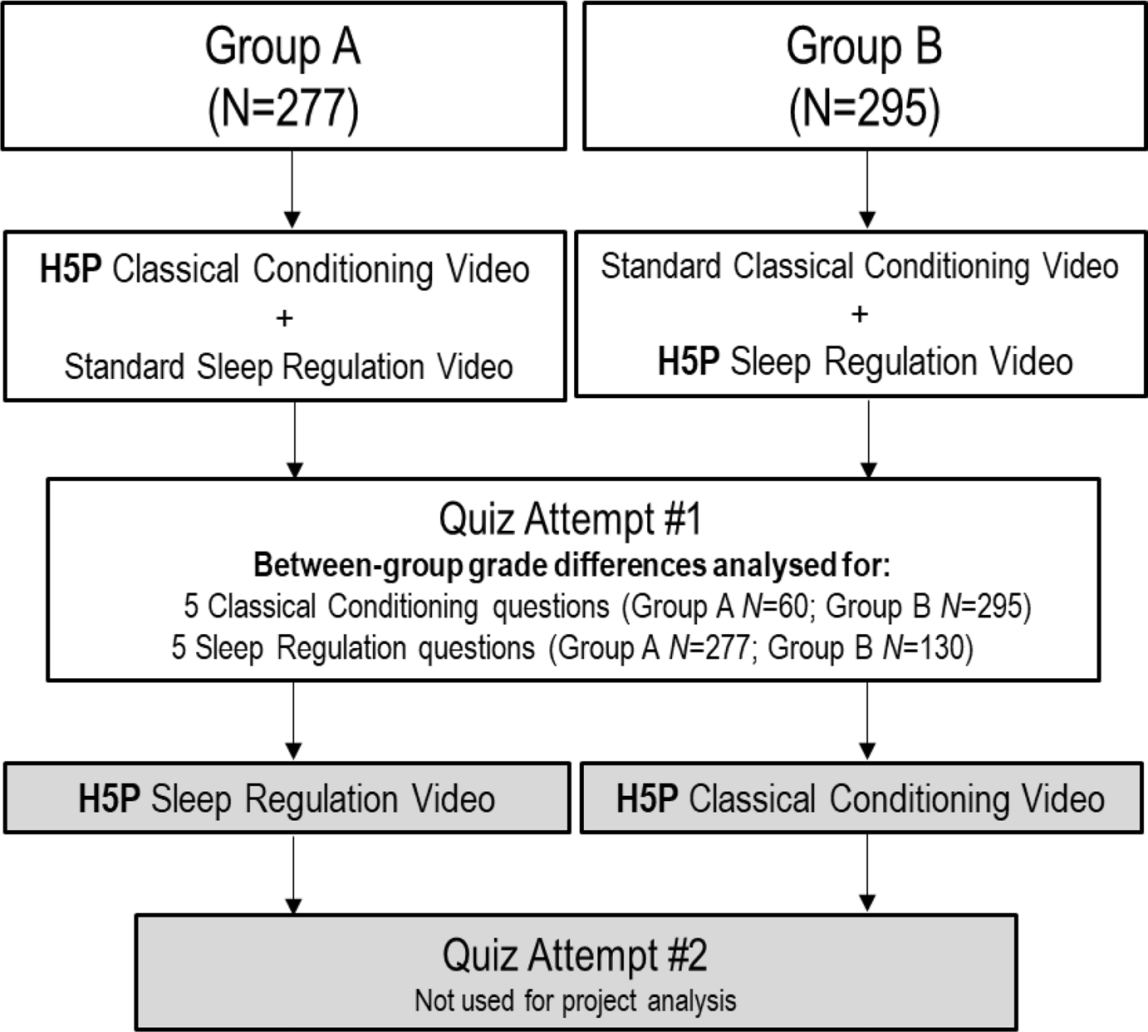
H5P Project Methodology. White boxes denote parts of the intervention used in the current study. N provided in the Quiz Attempt #1 box are those who completed all interactive components of the assigned H5P videos. Shaded grey boxes denote parts of the intervention used for the purposes of the course that were not included in the current study

To maintain student equity in terms of access to resources, a cross-over design was then implemented (Fig. 1). After students completed their first attempt on the summative assessment quiz, they were provided access to the interactive content video that they did not previously have access to. They were then allowed a second, final attempt on the summative assessment quiz. In other words, students in Group A who only had access to the original non-interactive Sleep Regulation video before their first summative assessment quiz attempt could then engage with the interactive version of the Sleep Regulation video before attempting the summative assessment quiz for a second time. The highest score counted to the students’ final grade. For the purposes of this study, only quiz results from the first attempt were taken into consideration.

#### Student feedback survey

After completing the summative assessment quiz, students were also asked to complete a feedback survey rating their experiences and perceptions on whether H5P interactive videos aided them in learning the course content. The survey consisted of 6 questions with response options on a 5-point scale ranging from ‘Strongly Disagree’ to ‘Strongly Agree’. An additional question on a Yes/No scale asked whether students thought it would be helpful for their learning if more online videos incorporated interactive elements. The survey was optional, and responses were anonymous. The subset of students from both conditions who opted to participate in the survey completed it after they had viewed the interactive videos and completed the summative assessment quiz.

#### Data analysis

Of those students who completed the summative assessment quiz, de-identified quiz scores for students in Group A who completed all of the H5P resources within the interactive Classical Conditioning content video (n = 60) were compared to students in Group B who did not have access to the H5P Classical Conditioning resource (n = 295). Similarly, the summative assessment quiz results of students in Group B who completed all of the H5P resources in the interactive Sleep Regulation content video (n = 277) were compared to students in Group A who did not have access to the Sleep Regulation H5P resource (n = 130). A one-way ANCOVA was conducted in SPSS (v.26), (IBM Corp, 2019) to determine between-group differences in quiz results, controlling for age and gender. Descriptive data are presented for the student feedback survey responses (n = 162).

## Results

Of 572 students, 190 (33%) watched the entire assigned video and completed all interactive elements. Of the students assigned to Group A (interactive H5P Classical Conditioning video and standard Sleep Regulation video; average age 31.4 ± 11.3y; 88% Females), a total of 60 students completed the interactive elements of the H5P video and 217 did not (21% of 277; Fig. 1). Of the students assigned to Group B (interactive H5P Sleep Regulation video and standard Classical Conditioning video; average age 31.3 ± 11.1y; 86% Females) a total of 130 completed the interactive elements of the H5P video and 165 did not (44% of 295). A *t-*test confirmed that there were no between-group differences in age distribution, *t*(120) = 1.3, *p* = 0.09. For the purposes of the analyses below, only students who completed all interactive elements of the assigned H5P video were included in comparisons with the alternate group who did not have access prior to the summative assessment quiz. Students who only partially completed the H5P interactive elements were excluded from the below analyses.

## Summative assessment quiz

**Fig. 2:**
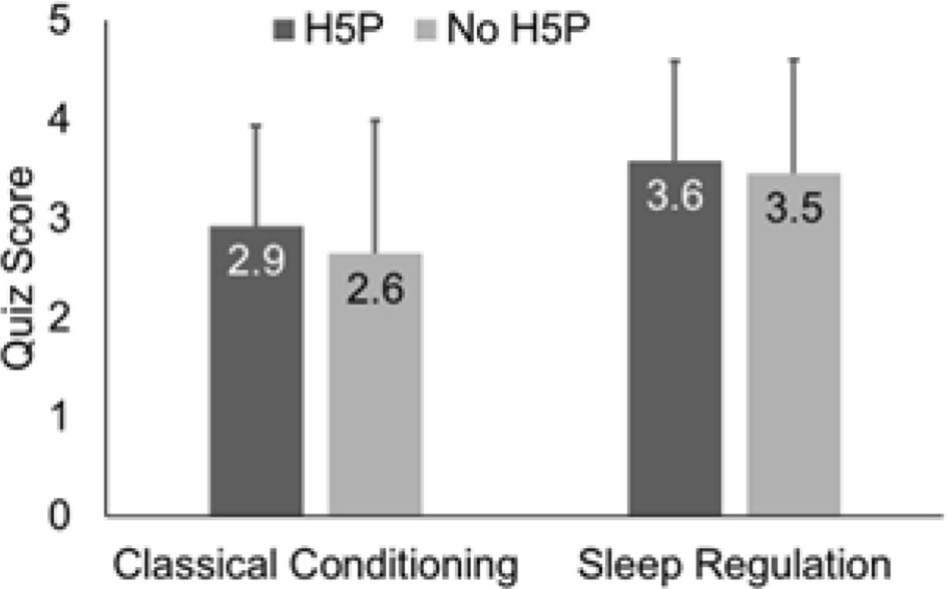
Mean quiz scores for five classical conditioning and five sleep regulation questions. Error bars represent standard deviation. Students who did not engage with the entire assigned H5P presentation were excluded from analyses

The possible total score for the five Classical Conditioning quiz questions and Sleep Regulation quiz questions ranged from 0–5 (1 mark per question). Mean total scores for the Classical Conditioning quiz questions and Sleep Regulation quiz questions are presented in Fig. 2 for Group A and Group B. When controlling for age and gender, there were no statistically significant differences between groups (H5P vs. no H5P) for the Classical Conditioning quiz results (*F*[1,186] = 0.02 *p* = 0.901) or Sleep Regulation quiz results (*F*[1,186] = 2.68, *p* = 0.103).

## Student feedback survey

**Fig. 3:**
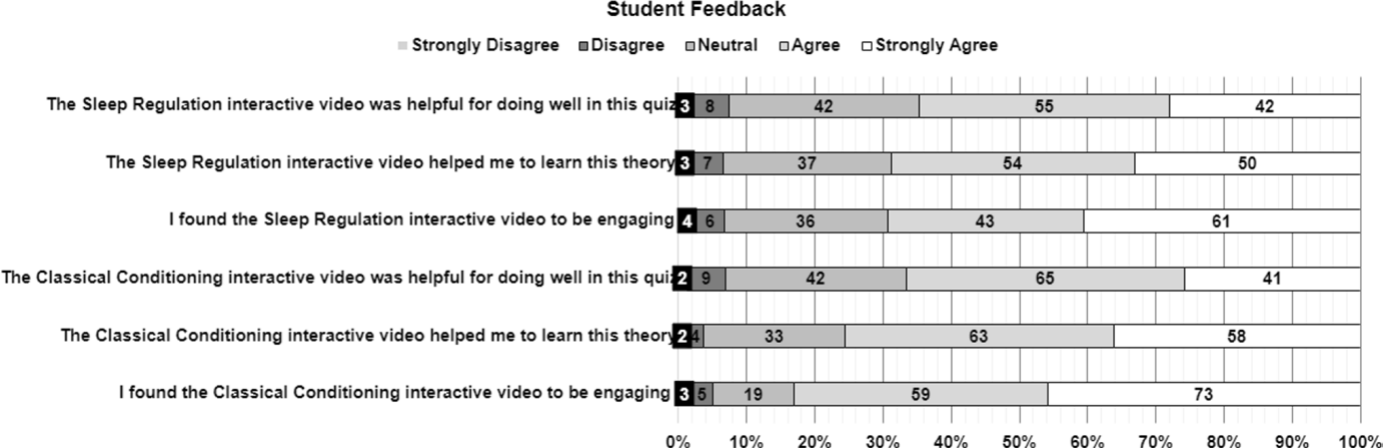
Student survey responses. Figures within each shaded bar denote number of students

The majority of students who completed the feedback survey indicated that the two H5P videos were engaging and assisted their learning and quiz results (Fig. 3). In addition, 97% of students indicated that they thought it would be helpful for their learning if more online videos incorporated interactive elements.

## Discussion

The results of this study show that participants exposed to H5P interactive resources did not have meaningfully improved summative quiz scores compared to students who did not have access to H5P. These results are in contrast to preliminary findings from Wilkie and Zakaria (2017) who found that H5P interactives let to improved pass rates.

This discrepancy in findings may be due to differences and limitations in our study design, as discussed below. Student survey feedback however, showed an overwhelmingly positive response to H5P resources. A total of 83% of respondents found the Classical Conditioning H5P resource engaging, while 67% provided positive responses indicating that the H5P resources were helpful for completing the summative assessment quiz. A total of 69% of respondents agreed that the Sleep Regulation H5P resource was engaging, and 65% indicated the H5P resources were helpful for the summative assessment quiz. Nearly all (97%) survey respondents indicated that they would like to see more interactive elements incorporated into videos to help with their learning. The findings of the current study align with previous studies that showed students perceived their content knowledge to be significantly improved after engaging with interactive H5P presentations (Sinnayah et al., 2021).

Williams (2006) found that student success rates were tied to availability of a variety of formative assessments and quality teacher-student interactions. The current study attempted to provide variety in formative assessments through the creation of the engaging H5P resources that were part of a suite of resources provided to students in the course. What was not necessarily present in the learning environment was sustained teacher-student interactions, as the H5P resources were developed to be primarily student driven, and analytics are not easily accessible to teaching staff. This means that while H5P resources may provide opportunities for students to reflect on their understanding of content and thus ask for assistance from the teaching team, there was still limited opportunity for teachers to implement a personalized teaching approach and engage in teacher-student interactions. Access to H5P analytics would have allowed teaching staff to provide feedback to students prior to the summative assessment, based on their results and interactions with the H5P resources. This limitation of the H5P plugin is problematic because evidence shows the importance of teacher presence, especially for online learning environments (Bawa, 2016; Williams, 2006).

In line with the constructivist approach, learning is seen as a social experience in which teacher presence forms an integral part of the learning environment (Park & Kim, 2020a, 2020b). This theory also highlights the work of Lai and Bower (2020) who found that educational technologies that focused on constructivist approaches were more effective in improving student learning outcomes. To enable effective teacher presence, online academics must be flexible and confident enough to facilitate students’ cognitive, affective and social requirements (Chi et al., 2018; Keengwe & Kidd, 2010; Poll et al., 2014; Thompson, 2006). This requires a level of technical capacity as well as institutional resources to effectively monitor and adapt to student needs. Although the current institutional Moodle set-up does not allow it, being able to effectively investigate the analytics of H5P resources would enable academics to be more flexible and adapt to student learning requirements. Finally, when interpreting the results in the context of the ICAP framework Chi and Wylie (2014), one would expect that the H5P resources, which provide an *Active* mode of engagement, would lead to improved student outcomes compared to the non-interactive *Passive* resources. However, this was not observed in the current study.

There are several methodological issues in this study that may explain the results and discrepancy with existing research. In a sample of 572 students, only a third of the cohort completed the interactive elements of their assigned H5P interactive video resource prior to their first quiz attempt. This could be due to limitations in how the H5P interactive videos were presented on the Moodle page. To ensure an experimental-control group setting which restricted access of videos depending on the assigned group, it was not possible to embed the H5P interactive video resources using the *Label* function of Moodle. This meant that students had to click on a hyperlink to access the videos, which may not have been apparent to them, as their experience is that all video content is embedded on the page.

Furthermore, only five questions in the quiz evaluated the learning outcomes of each of the H5P interactive videos. The rest of the questions in the quiz measured student understanding of other content presented in the weekly subtopics (for example, operant conditioning, structure of sleep, etc.). Another limitation of this study was the amount of time between the H5P interventions (week 4 and week 5) and the summative assessment quiz (week 7). This was unavoidable due to the constraints of course requirements. Therefore, there could have been a range of external factors, such as poor follow up study strategies, impacting on student scores. Future iterations should look to expand the interactive content in each weekly subtopic and the number of assessed quiz questions, as this would provide a better indication of student outcomes. A strength of this study was the implementation of a survey to gauge students’ subjective experiences of the H5P resources. The survey results indicate that students found that H5P had helped them to understand the content better and to perform better in the quiz. The positive responses to the H5P resources support the cognitive perspective that calls for learning that is student centric, personalized and encourages self-regulation (Chatti et al., 2010; Doherty & Blake, 2010; Ellis & Goodyear, 2013), as this promotes deeper engagement with the content. The fact that the intervention improved subjective experiences of the learning content is promising given that satisfaction and positive student attitudes towards learning are related to academic achievement and student retention (Oja, 2011; Topal, 2016).

Another consideration is that students may not have the necessary digital literacy skills to successfully engage in interactive resources, even if they are provided in online courses. Keppell, (2014) points out that higher education institutions may not be properly scaffolding digital literacy skills, and may be making the assumption that students already have these skills. For example, a recent study by Šorgo et al. (2017) on Slovenian university students found that being a ‘digital native’ with assumed ‘natural’ capabilities in information and communications technology (ICT) tools, is a poor predictor of information literacy skills that enable academic competencies. These digital literacy skills may also need to be developed in older students who have extensive working experience but lack proficiency in ICT tools (Geri et al., 2017). In this study, there were students from 27 different degree programs and the age range was from 18 -78 years, reflecting a diverse student cohort. What this potentially means is that students may not be accessing the content from the same level of digital literacy, and thus may have needed further support in navigating interactive resources and the online environment. Furthermore, a large number of students were coming from on-campus degree programs that use a different Moodle platform; thus the unfamiliarity could have impacted interaction rates. Providing students with a rationale for why they should engage with interactive resources may also improve interaction rates. Demographics such as year of study, mode of study, and educational background were not readily available for the current study. Examining these variables more closely would be useful in further research on engagement with H5P activities and student outcomes. Given the positive experiences of students who used the H5P resources in this study, investigating ways to promote use of interactive resources is also likely a valuable priority. Additional studies on whether H5P improves grades would add further weight to recommendations to use H5P resources.

## Conclusions

The aim of this study was to investigate how H5P can contribute to the overall learning experience of online course content and to determine whether the intervention of interactive video features improved student learning outcomes. The results of the study were mixed in that student academic outcomes did not improve when exposed to H5P. This may have been due to limitations in accessibility and the timing of the H5P intervention in relation to the summative assessment quiz. However, feedback indicated that students found the interactive elements of H5P to be positive and that they would like to see interactive videos in more courses. This study adds to the limited literature regarding the effectiveness of H5P resources in online tertiary courses. Future studies should aim to improve on the methodological issues discussed above to provide a more accurate measure of the effectiveness of H5P resources. Furthermore, should future iterations of Moodle improve how H5P analytics are presented to the teaching team, this would enable more nuanced teacher-student interactions through targeted feedback opportunities.

## Figures and Tables

**Table 1:**
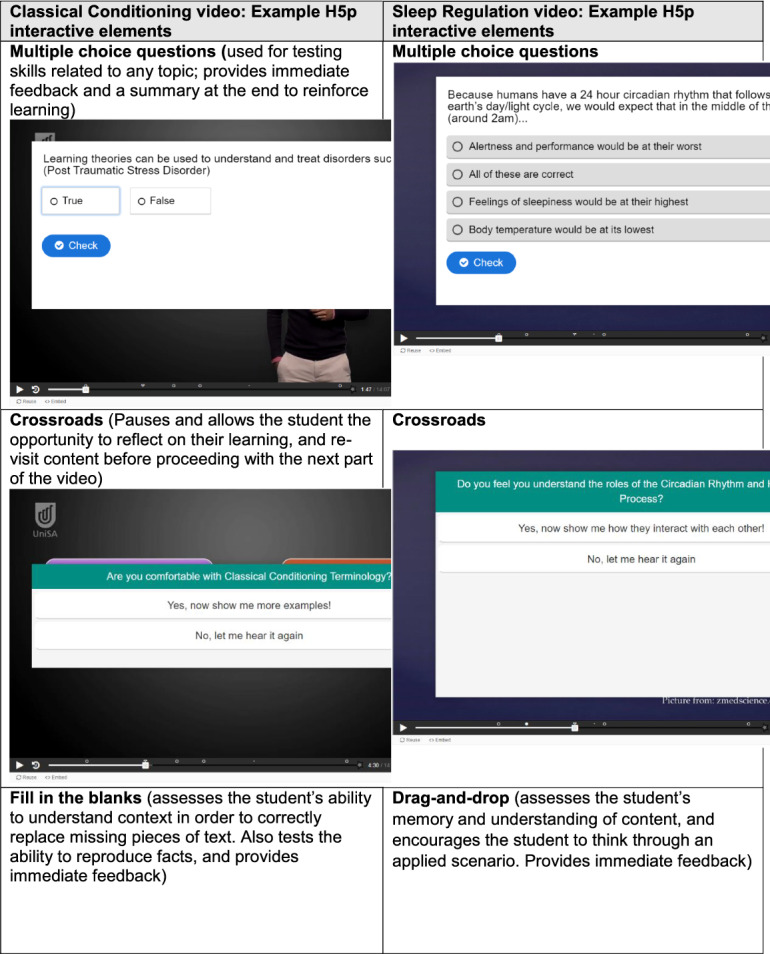

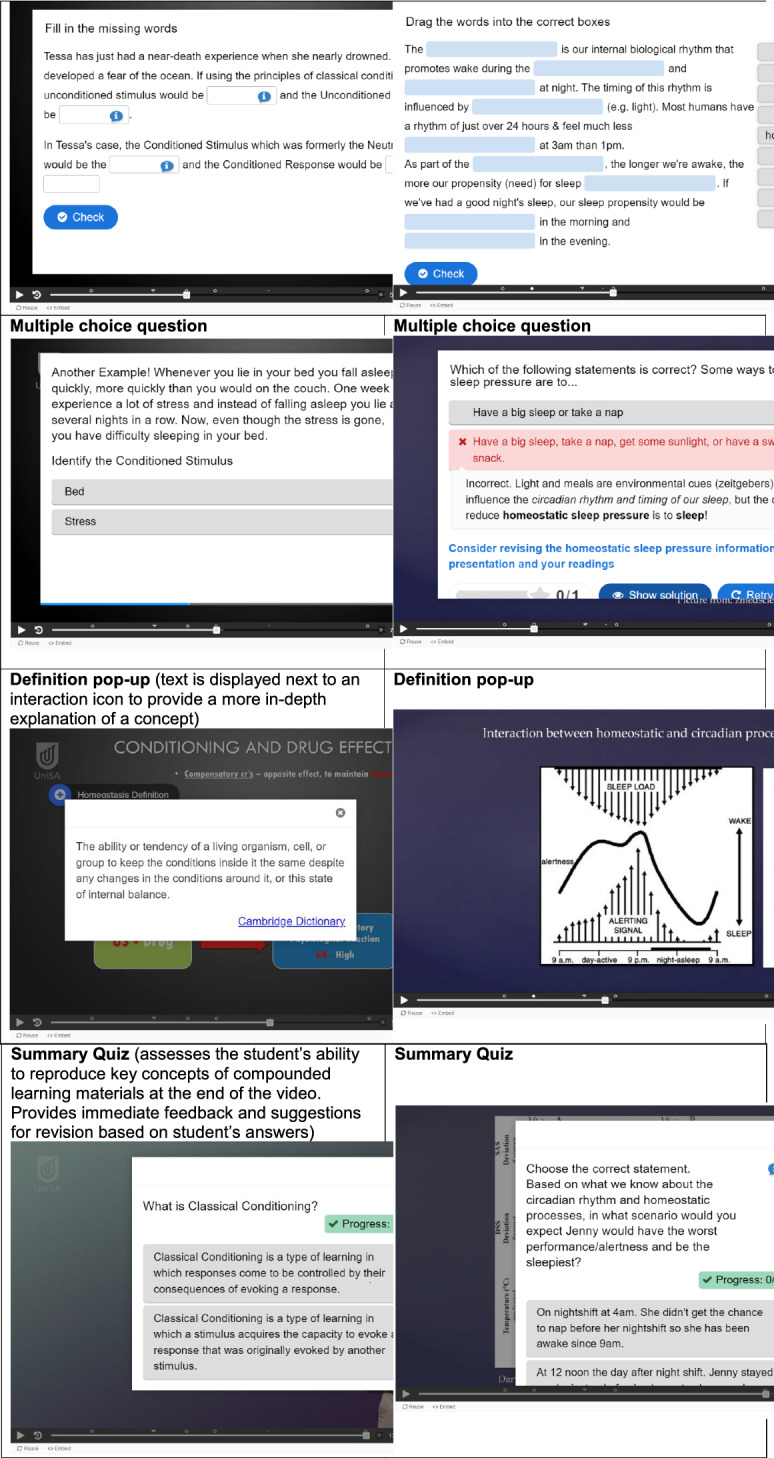
Examples of H5P interactive elements embedded into the online videos

## Appendix

See Table 1.
